# Supplementary material for: Femtosecond X-ray induced changes of the electronic and magnetic response of solids from electron redistribution
Source: Nat Commun. 2019 Nov 21;10:5289. doi: 10.1038/s41467-019-13272-5 (PMC6872582; doi:10.1038/s41467-019-13272-5)
Supplement: Supplementary file 1 — Supplementary Information [file 41467_2019_13272_MOESM1_ESM.pdf]

Femtosecond X-ray induced changes of the electronic and magnetic response of solids from electron redistribution

Higley et al.

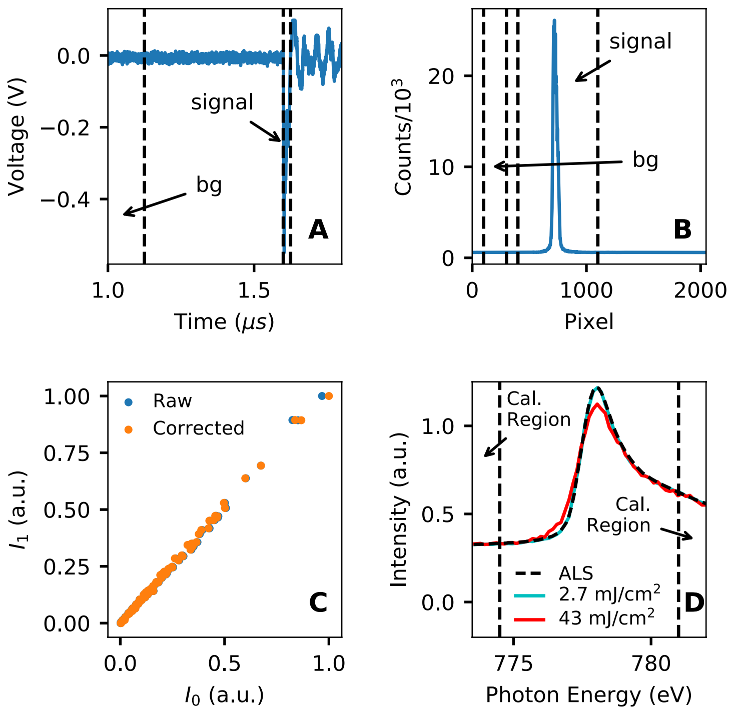

Supplementary Figure 1: Conversion of raw data to X-ray absorption spectra. (A) Waveform generated by MCP I_0 detector in response to an X-ray pulse. An I_0 signal for each pulse was calculated from these waveforms as the average of the signal region minus the average of the background (bg) region. To account for any signal in the absence of X-rays, the average of this I_0 signal recorded without X-rays was subtracted from each I_0 signal recorded with X-rays. (B) Values read out from downstream I_1 CCD detector for a single X-ray pulse. The detector was operated in one-dimensional mode, and thus the detector reads out one value for each pixel column. The same procedure as for the I_0 detector was applied to get the I_1 signal for each X-ray pulse. (C) Scatter plot of I_0 versus I_1 signals before and after correction for a slight I_0 nonlinearity at the highest intensities. (D) X-ray absorption spectra (XAS) recorded in the low fluence limit at the Advanced Light Source (ALS) synchrotron light source and those recorded for a few different fluences at the Linac Coherent Light Source (LCLS). The XAS is calculated as the negative logarithm of I_1/I_0 minus a background with a linear dependence on the photon energy. The linear background is calculated so that the XAS spectra align with the ALS spectra in the calibration regions, which are well away from the absorption resonance. Source data provided as a source data file.

	Co	$(Co_{0.5}Pt_{0.6})$x11 (thicknesses in nm)
C_p [10^6 Jm^{-3}K^{-1}]	2.07	2.98
γ [Jm^{3}K^{-2}]	665	720
g_{ep} [10^{16} Js^{-1}m^{-3}K^{-1}]	405	264

T_C [K]	1388	550
R [10^{12} s^{-1}]	25.3	9.396
Atoms per cubic nm (average)	90.5	77.4

Supplementary Table 1: Microscopic three temperature model parameters. Parameters were obtained from literature values [1–3].

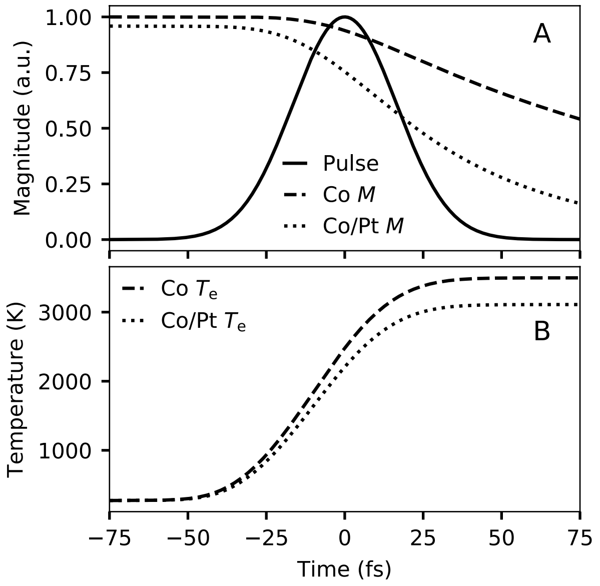

Supplementary Figure 2: Simulated optically induced demagnetization. The degree of demagnetization was calculated for Co and Co/Pt with a 39 fs FWHM optical pulse which deposits 280 meV total per atom (corresponding to 140 meV pulse averaged absorbed energy). (A) Intensity profile of incident optical pulse and sample magnetization as a function of time. (B) Electronic temperature as a function of time. Source data provided as a Source Data file.

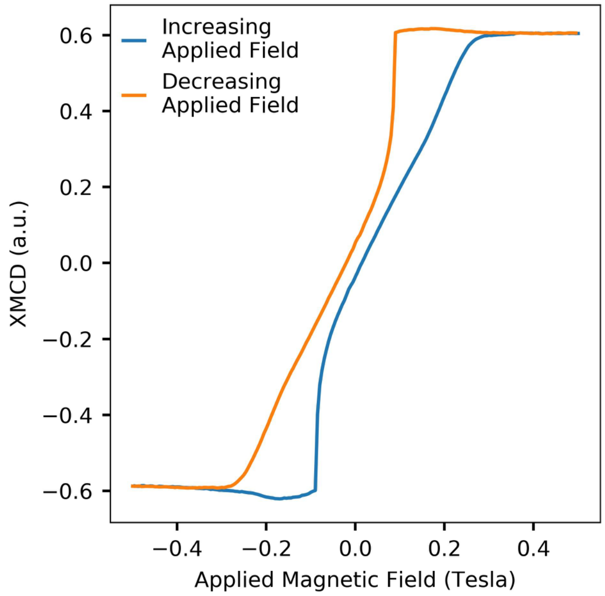

Supplementary Figure 3: Hysteresis loop of Co/Pd sample. The data was recorded in electron yield mode at the Advanced Light Source synchrotron light source. For each magnetic field point, the XMCD (X-ray Magnetic Circular Dichroism) was calculated as the difference of the X-ray absorption intensity with opposite orientations of X-ray circular polarization. Source data provided as a Source Data file.

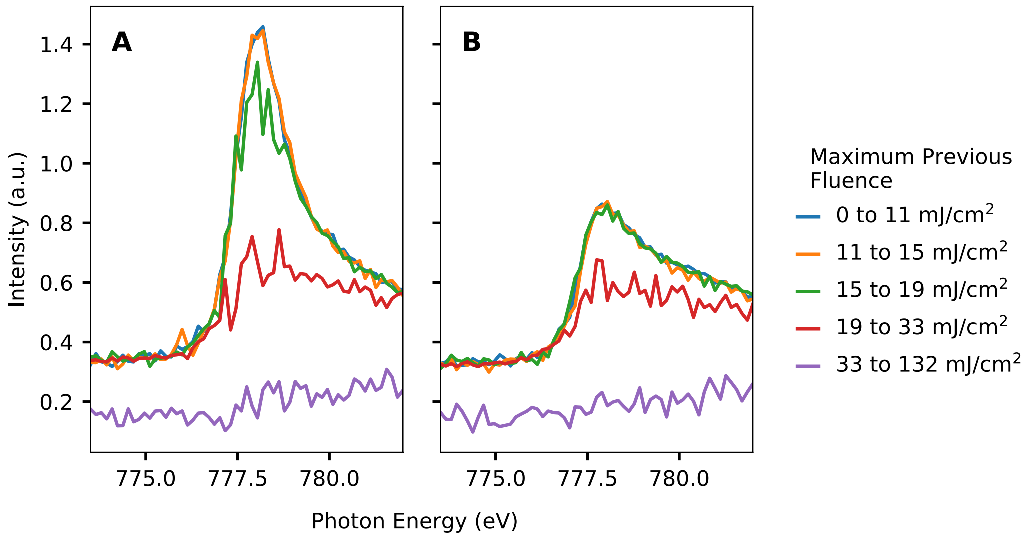

Supplementary Figure 4: X-ray induced damage of Co/Pd. X-ray absorption spectra are shown as a function of the maximum previous incident X-ray fluence which the sample has been exposed to. (A) shows the case for parallel (***σ***$_-$) orientation of X-ray polarization and sample magnetization, while (B) shows the case for anti-parallel (***σ***$_+$) orientation of X-ray polarization and sample magnetization. Source data provided as a Source Data File.

Supplementary References

[1] Koopmans, B. et al. Explaining the paradoxical diversity of ultrafast laser-induced demagnetization. Nat. Mater. **9**, 259–265 (2010).

[2] Bergeard, N. et al. Hot-electron-induced ultrafast demagnetization in Co/Pt multilayers. Phys. Rev. Lett. **117**, 147203 (2016)

[3] Lide, D. R. *CRC handbook of chemistry and physics* (CRC press, 2005).
